# Spin-Adapted Restricted Open-Shell Hartree–Fock and Its Dynamic Correlation Extension

**DOI:** 10.1021/acs.jctc.6c00379

**Published:** 2026-05-15

**Authors:** Maru Song, Luca Bonfirraro, Ignacio Fdez. Galván, Roland Lindh, Giovanni Li Manni

**Affiliations:** † 28326 Max Planck Institute for Solid State Research, Stuttgart 70569, Germany; ‡ Department of Chemistry for Life Sciences, 8097 Uppsala University, P.O. Box 576, Uppsala 75123, Sweden

## Abstract

We report a spin-adapted configuration-state-function restricted open-shell Hartree–Fock implementation in OpenMolcas, hereafter denoted CSF-ROHF. The implementation is based on the Graphical Unitary Group Approach for reduced density matrix evaluation, and on the Generalized Active Space and super-configuration interaction algorithms for the orbital optimization. The method enables orbital optimization of a single spin-pure electronic configuration at mean-field cost. Analysis of CSF-ROHF convergence reveals that the initial orbital ordering is decisive for avoiding and escaping local minima during optimization. The computational efficiency of the method, in terms of both iteration count and wall time, is demonstrated on {[Ni^II^​​(H_2_O)_4_]_*n*_O_*n*–1_​​(H_2_O)_2_}^2+^ ​​(*n* = 1, ..., 10) model systems. For spin gaps of iron–sulfur clusters, CSF-ROHF exhibits intrinsic limitations yielding qualitatively incorrect gaps and offering minimal-to-no advantages over conventional high-spin ROHF. To address these limitations, we introduce a near-mean-field-cost orbital optimization protocol that incorporates dynamic correlation via a second-order, spin-adapted perturbation strategy relying on the recently developed Stochastic-SplitGAS algorithm. The resulting perturbatively corrected state representations are substantially improved relative to the bare CSF-ROHF results, yielding energies and spin gaps in excellent agreement with far more expensive Complete Active Space Self-Consistent Field calculations.

## Graphical abstract

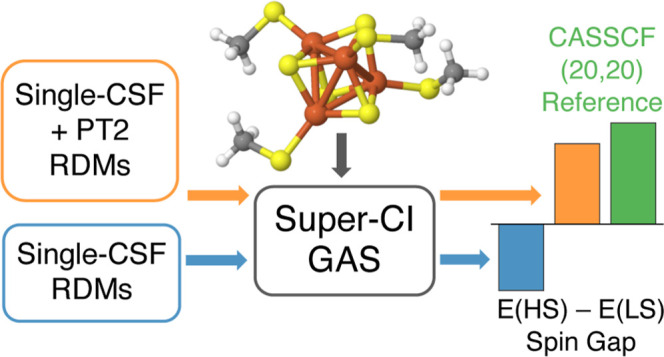

## 1 Introduction

Unpaired electrons in molecules and materials give rise to a variety of interesting properties, including catalytic activity of transition metal clusters,^1–3^ magnetism,^4^ their use as platforms for quantum bits,^5^ and characteristic spectroscopic signatures ​​(e.g., electron paramagnetic resonance).^6–9^ Accurate theoretical descriptions of such phenomena require treating strong electron correlation ​​(*Class 2* systems in ref 10) for the many unpaired electrons in their low-energy states, thus, necessitating advanced multiconfigurational approaches^11^ for qualitatively correct descriptions. Multiconfigurational wave functions are typically obtained via the complete active space self-consistent field ​(CASSCF) method.^12–17^ In CASSCF, the many-body wave function is built by distributing the active electrons in the active orbitals in all possible ways compatible with spin and space symmetry, while the orbitals are variationally optimized with respect to the energy of the multiconfigurational wave function.

The exponential scaling of the configuration interaction ​(CI) expansion with respect to the size of the active space severely limits the applicability of conventional CASSCF algorithms to approximately 18 electrons and 18 orbitals.^18^ This limitation is alleviated in modern electronic structure methods by replacing the conventional CI solver ​(Davidson diagonalization,^19,20^ within the direct-CI algorithm^21–23^ ) with approximate CI eigensolvers, such as density matrix renormalization group,^24–28^ full-CI quantum Monte Carlo ​(FCIQMC),^29–32^ and selected-CI.^33–40^ The combination of FCIQMC, as CI solver and Super-CI, as orbital optimizer, has enabled Stochastic-CASSCF calculations for much larger active spaces.^41–44^ An alternative strategy is to partition the active space into smaller subspaces and impose restrictions on the interspace electron excitations, as realized in restricted active space ​(RAS),^23^ Occupation-Restricted Multiple Active Space ​(ORMAS),^45^ and generalized active space ​(GAS).^46^ The recently developed Stochastic-GAS algorithm^47,104^ integrates the two strategies, facilitating the efficient treatment of large active spaces.

At the practical level, restricted open-shell Hartree–Fock ​(ROHF)^48–54^ orbitals—optimized for the high-spin ​(HS) state—have generally been used as starting orbitals for many-unpaired-electron systems to reduce the number of multiconfigurational SCF ​(MC-SCF) iterations and avoid local minima. Although HS-ROHF generally provides a well-defined starting state—free of spin contamination and typically converging reliably—optimizing orbitals for the target spin state can, in principle, further facilitate convergence in subsequent post-HF methods, including MC-SCF calculations. Recently, generalized ROHF algorithms have been introduced,^55,56^ enabling mean-field-cost orbital optimizations for a single configuration state function ​(CSF). We refer to these approaches as CSF-ROHF, following the terminology introduced in ref 55.

Despite the algorithmic advance of the two suggested strategies, both works have clearly shown that CSF-ROHF optimizations without adequate post-ROHF treatments perform poorly, yielding to qualitatively incorrect spin gaps. Crucially, this deficiency in the energetics has long been recognized as arising from the absence of ionic ​(through-space charge-transfer [CT]) configurations across the magnetic centers.^57–60^ It also remains unclear whether CSF-ROHF orbitals specifically optimized for the targeted spin state provide an advantage over conventional HS-ROHF orbitals in subsequent MC-SCF treatments, in terms of iteration count, wall-clock time and in avoiding potential local minima during the optimization. These considerations motivate two central questions:

1. How can missing CT effects be effectively incorporated into CSF-ROHF orbital optimizations while keeping the near-mean-field cost?
2. Under what circumstances do CSF-ROHF orbitals offer a tangible advantage over conventional HS-ROHF?

Furthermore, CSF-ROHF algorithms can suffer from technical limitations as they can be slower to converge as compared to the corresponding HS-ROHF,^55^ and sensitive to local minima.^56^ Therefore, it is also important to understand the convergence behavior of CSF-ROHF optimization.

1. Are local minima a recurring issue in CSF-ROHF optimizations, and if so, how can they be avoided?
2. Is the significantly slower convergence of the reported CSF-ROHF algorithm^55^ an intrinsic feature of the method or can it be mitigated through improved algorithms and better initial guesses?

To address these questions, we implemented a CSF-ROHF method within the OpenMolcas quantum chemistry software package,^61^ that relies on the Graphical Unitary Group Approach ​(GUGA) for reduced density matrix evaluation, on the Super-CI algorithm^62–64^ for the orbital relaxation, and the GAS algorithm to enable orbital rotations within the active space.^46^

While both the previously reported and our CSF-ROHF algorithms can utilize an arbitrary genealogically coupled CSF, they differ in their underlying theoretical formulations. The work of Gouveia et al.^55^ is based on the ROHF formulation of Edwards and Zerner,^53^ with the open-shell Fock matrix expression of Fernández Rico et al.^65^ Burton^56^ develops a geometric direct minimization for a single CSF using the analytic form of the single-CSF Hessian. Our CSF-ROHF implementation instead minimizes the energy of a single CSF via the Super-CI algorithm^62–64^ within the generalized Fock matrix framework.^11,14^ As the approach of ref 56 is specifically tailored for single-CSF orbital optimization, it may offer advantages in convergence behavior. In contrast, our formulation within the generalized Fock matrix framework readily enables extensions beyond the single-CSF level, in particular the incorporation of dynamic correlation effects, which constitutes the main focus of the present work.

Throughout this paper, CSF-ROHF denotes our implementation unless otherwise stated. The theoretical background and implementation details of CSF-ROHF are outlined in Section 2, and computational details for all testcase calculations are provided in Section 3. The convergence behavior of CSF-ROHF as a function of the starting orbitals is analyzed in some details for the nitrogen molecule at a bond distance of 3 Å ​(Section 4.1), while its computational efficiency and scaling is assessed in terms of iteration count to convergence and wall time, for the linear {[Ni^II^​(H_2_O)_4_]_*n*_O_*n*–1_​(H_2_O)_2_}^2+^ ​(*n* = 1, ..., 10) model systems ​(Section 4.2).

We address the limitations of the method in terms of incorrect energetics and lack of advantages over HS-ROHF orbitals for subsequent MC-SCF treatments by introducing a near-mean-field-cost extension of CSF-ROHF that incorporates dynamic correlation effects based on second-order, spin-adapted Löwdin downfolding^66,67^ during the orbital optimization. This extension is enabled by the recently developed Stochastic-SplitGAS ​(SSG) algorithm,^68^ which allows for a general uncontracted second-order perturbation theory both in Slater determinant and CSF bases. The combined approach is denoted as SSG-CSF-ROHF, and its applicability to all collinear low-spin ​(LS) states of [Fe­(III)_2_​S_2_​(SCH_3_)_4_]^2−^, [Fe(III)_4_​S_4_​(SCH_3_)_4_], and the nitrogenase P-cluster, is assessed in Section 4.3.

As the focus of this work is the development of a single-CSF method, we restrict our study to collinear spin states, for which a single GUGA-CSF provides an adequate and physically meaningful description. In this context, collinear states refer to spin configurations in which the local spins ​(at the magnetic centers) are aligned either parallel or antiparallel across different magnetic sites. Such collinear states commonly correspond to the ground states of antiferromagnetic systems^3,44,69^ and play a central role in determining their magnetic properties.

In contrast, noncollinear states are characterized by intermediate spin coupling across the metal centers. These states are intrinsically multiconfigurational within the GUGA genealogical spin-coupling representation ​(see details in ref 70), requiring multiple GUGA-CSFs to represent the corresponding CAS-CI eigenvectors. As a consequence, they cannot be represented within a single-CSF ansatz, even when perturbative corrections are included, and therefore lie outside the scope of the present approach.

Section 5 concludes this work and discusses future perspectives.

## 2 Theory and Implementation

In this section, we briefly review the theoretical concepts relevant to the CSF-ROHF method and its extension for perturbatively incorporating dynamic correlation effects. The presentation focuses on the essential aspects required to understand our implementation and the subsequent discussion of results. For comprehensive introductions to the individual topics, we refer the reader to the references cited in each subsection. An overview of the CSF-ROHF implementation is also provided.

### 2.1 Spin-Adapted Basis in GUGA Framework

The unitary group approach and its graphical extension, GUGA,^71–78^ provide a framework for systematically constructing genealogical CSFs and representing the electronic Hamiltonian in a spin-adapted basis. For an electronic state with *N*_e_ electrons, *N*_o_ spatial orbitals, and total spin *S*, each CSF is uniquely defined by a sequence of orbital couplings. Empty, singly occupied with spin-up or spin-down coupling, and doubly occupied orbital are denoted by the symbols 0, *u*, *d*, and 2, respectively ​(Table 1).

**Table 1:** Orbital Coupling Symbols and Their Corresponding Changes in Electron Number ​(Δ*N*_e_) and Spin ​(Δ*S*)

coupling	Δ*N*_e_	Δ*S*
0	0	0
*u*	1	1/2
*d*	1	−1/2
2	2	0

The complete spin-adapted basis for a given ​(*N*_e_, *N*_o_, *S*) consists of all coupling sequences satisfying

1 $$\sum_{i=1}^{N_{o}}\Delta N_{e}^{\left(i\right)}=N_{e},,\sum_{i=1}^{N_{o}}\Delta S^{\left(i\right)}=S$$

subject to the constraint

2 $$\sum_{i=1}^{k}\Delta S^{\left(i\right)}\geq 0,\forall k\in \left[1,N_{o}\right]$$

where Δ*N*_e_^​(*i*)^ and Δ*S* ^​(*i*)^ denote the changes in electron number and spin associated with the *i*-th orbital coupling. As an illustrative example, a system with ​(*N*_e_, *N*_o_, *S*) = ​(2, 2, 0) has three valid CSFs, {|*ud*⟩, |20⟩, |02⟩}. The coupling sequence |*du*⟩ is not permitted, as the partial sum $\sum_{i=1}^{1}\Delta S^{\left(i\right)}=-\frac{1}{2}$ violates the partial spin constraint ​(eq 2).

### 2.2 Löwdin Partitioning, SplitGAS and Its Stochastic Implementation

Within Löwdin’s downfolding,^66,67,79,80^ a many-body wave function is partitioned into a principal space, P , typically consisting of the most important electronic configurations ​(highest weights) and a perturber space, Q , containing nondominant electronic configurations, responsible for what is commonly referred to as dynamic electron correlation. Accordingly, the corresponding CI Hamiltonian matrix is partitioned into four blocks, PP , PQ , QP and QQ . An effective Hamiltonian, **H̃**, of PP dimensionality is then constructed whose resolution leads to the eigenstates in P , with corrections from the full eigenvalue problem. Within the SplitGAS method,^68,81,200^ the effective Hamiltonian expression is truncated after the second term, effectively yielding to a second order perturbative correction

3 $${\mathrm{H̃}}_{ij}=H_{ij}+\sum_{\alpha \in Q}\frac{H_{i\alpha }H_{\alpha j}}{E-H_{\alpha \alpha }}$$

The partitioning of the wave function in P and Q , is obtained via two disjoint sets of GAS constraints.^46^ From eq 3 of SplitGAS, the diagonal approximation in the QQ block is apparent ​(Figure 1).

**Figure 1:**
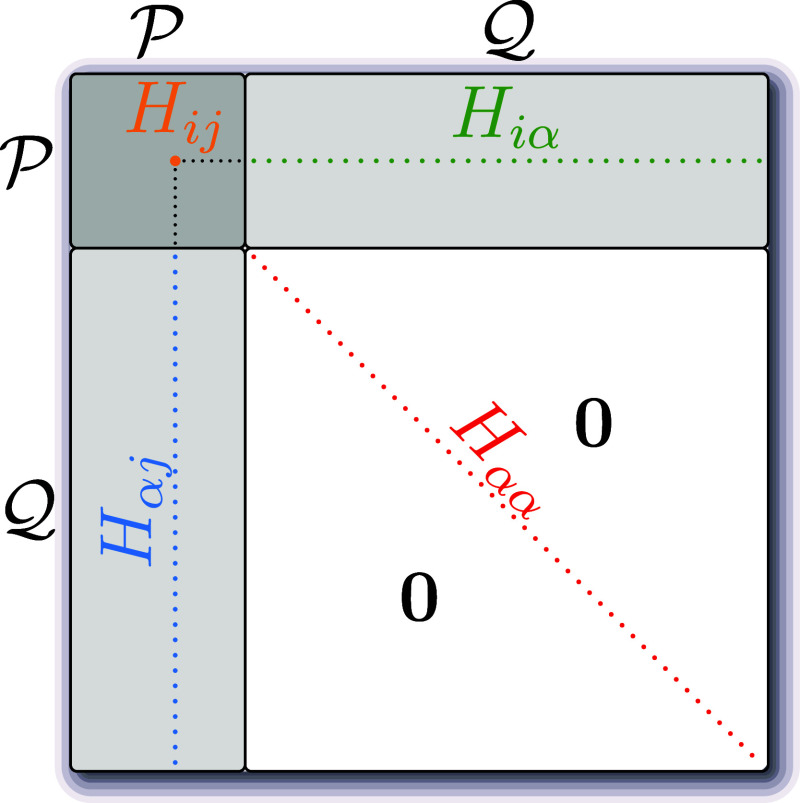
Schematic structure of the effective SSG Hamiltonian matrix.

In the original nonstochastic SplitGAS variant,^81^ the effective Hamiltonian was built directly in the construction of the *sigma* vector ​(of size P ) within the direct-CI formulation. Whereas in the recent SSG framework,^68^ the structure of the full Hamiltonian matrix is retained, and all off-diagonal elements in QQ are considered as zeroes ​(by construction) and never sampled at the spawning step of the stochastic wave function optimization.

In the context of CSF-ROHF, the P space of SSG-CSF-ROHF is reduced solely to the target CSF, with the Q space consisting of all coupled CSFs, serving as perturbative correction to the single CSF. While the number of coupled configuration to the single CSF in the P space is generally small and the problem could be solved nonstochastically in many cases, we directly demonstrate the applicability of the stochastic variant, which could be of great interest for large systems and/or one electron basis sets.

For metal-centered active spaces, as the ones discussed in Section 4, the correction introduced by SSG mostly accounts for through-space CT configurations ​(one-electron hopping configurations) as well as exchange terms ​(two-electron excitation process). The latter, however, might become vanishingly small within the context of Quantum Anamorphosis.^70,82–84^

### 2.3 CSF-ROHF Implementation

Our implementation is motivated by viewing CSF-ROHF as a special case of a MC-SCF method, in which the generalized Fock matrix^11,14^

4 Fnm=∑qDmqhnq+∑qrsdmq,rs gnq,rs

is constructed from the reduced density matrices ​(RDMs) of a reference wave function and used to optimize the orbitals. Here, all indices run over spatial orbitals, and *h*_nq_ and *g*_nq,rs_ denote the one- and two-electron integrals, respectively. The one- and two-electron RDMs for a wave function |Ψ⟩ are defined as

5 $$D_{pq}=\langle \Psi |{Ê}_{pq}|\Psi \rangle$$

6 $$d_{pq,rs}=\langle \Psi |{ê}_{pq,rs}|\Psi \rangle$$

where ${Ê}_{pq}=\sum_{\sigma }{â}_{p\sigma }^{†}{â}_{q\sigma }$ and ${ê}_{pq,rs}={Ê}_{pq}{Ê}_{rs}-\delta_{qr}{Ê}_{ps}$ . The operator *â*_*p*σ_^†^ creates a spin-σ electron in spatial orbital *p* and its Hermitian conjugate annihilates a spin-σ electron from the same orbital.

This generalized framework allows to build Fock matrices for any types of reference wave functions. Consequently, CSF-ROHF can be implemented within the multiconfigurational framework, and dynamic correlation effects can be incorporated without altering the underlying SCF algorithm. Below, we describe the two key modifications required for CSF-ROHF implementation and then outline its extension to the dynamically correlated SSG-CSF-ROHF scheme.

​(i) Constructing single-CSF Fock matrix. In eq 4, the configurations of the reference wave function enter exclusively through the RDMs. For CSF-ROHF, the reference is a single CSF, so only single-CSF RDMs are required. In this case, only diagonal matrix elements of eqs 5 and 6 contribute, leading to a considerable simplification in which only nonvanishing terms remain. The one-RDM reduces to
7 $$D_{pp}=\langle CSF|{Ê}_{pp}|CSF\rangle =n_{p}$$
  where *n*_*p*_ is the occupation number of orbital *p*. The nonvanishing two-RDM elements are
8 $$d_{pp,qq}=\langle CSF|{ê}_{pp,qq}|CSF\rangle =n_{p}n_{q}-\delta_{pq}n_{p}$$
9 $$d_{pq,qp}=\langle CSF|{ê}_{pq,qp}|CSF\rangle =\langle CSF|{Ê}_{pq}{Ê}_{qp}|CSF\rangle -n_{p}$$
  The exchange terms ​(*d*_*pq,qp*_) can be further simplified using GUGA, as derived in Appendix A.2.1 of ref 85. Because the reference consists of a single configuration, no CI coefficients appear in the RDM expressions. The RDMs are therefore fixed throughout the orbital optimization and need to be evaluated only once at the beginning of the calculation.
​(ii) Enabling intra-active orbital rotations. In CASSCF, orbital rotations within the active space are redundant, as the orbital degrees of freedom are already captured by the FCI expansion within the active space; thus, these rotations are generally excluded during optimization, and only rotations between different subspaces are considered ​(inactive–active, inactive−virtual, active−virtual). On the contrary, within the GASSCF framework, the active space is partitioned into multiple orbital subspaces, with occupation constraints restricting excitations between them.^46^ As a result, orbital rotations between distinct GAS subspaces are only partially redundant and are to be included in the orbital optimization. The CSF-ROHF is an extreme case of GAS, where the wave function in the active space is reduced to a single CSF, and orbital rotations within the active space are nonredundant and must be included in the optimization. Thus, in CSF-ROHF we exploit the GAS flexibility of allowing intra-active-space rotations, and assign each GAS subspace to shells of the target CSF—shell refers to a set of orbitals with identical consecutive *u* or *d* coupling—to enable orbital rotations within the active space.

These two modifications permit a straightforward implementation of CSF-ROHF in OpenMolcas,^61^ where GASSCF has been implemented as extension of the RASSCF module.

For the SSG-CSF-ROHF extension, dynamic correlation effects are incorporated into the RDMs directly from the SSG wave function. The SSG-RDMs are then used to construct the Fock matrix. To remain consistent with CSF-ROHF, the SSG-RDMs are kept fixed during orbital optimization. Although updating the SSG-RDMs at every iteration is possible since they depend on both the evolving orbitals and the CI coefficient optimization, preliminary tests indicate that such updates have little effect on the final optimized orbitals for the systems here investigated.

Table 2 summarizes the key differences among CASSCF, CSF-ROHF, and SSG-CSF-ROHF. The only technical difference between CSF-ROHF and SSG-CSF-ROHF is the type of RDMs used—single-CSF RDMs in the former and SSG-RDMs in the latter.

**Table 2:** Comparison between CASSCF, CSF-ROHF, and SSG-CSF-ROHF^a^

	CASSCF	CSF-ROHF	SSG-CSF-ROHF
basis	complete^a^ CI	single CSF	single CSF in P
RDM type	multiconfigurational	single CSF	single CSF + PT2^b^
RDM update	updated every iteration^c^	fixed	fixed
active–active rotations	not necessary	inter-GAS only	inter-GAS only

a Complete within spin and spatial symmetry restrictions.

b Obtained from the SSG calculation.

c To incorporate updated CI coefficients.

### 2.4 The Super-CI Orbital Optimization

Orbital optimization in CSF-ROHF is performed using the Super-CI method,^62–64^ as implemented in OpenMolcas.

The stationarity condition for a multiconfigurational reference state |REF⟩ is given by the generalized Brillouin theorem

10 $$\left\langle REF\left|\left[Ĥ,{Ê}_{pq}-{Ê}_{qp}\right]\right|REF\right\rangle =0$$

which can be expressed in terms of the generalized Fock matrix as

11 $$\left\langle REF\left|\left[Ĥ,{Ê}_{pq}-{Ê}_{qp}\right]\right|REF\right\rangle =2\left(F_{pq}-F_{qp}\right)$$

Equation 10 represents the necessary and sufficient condition for a local minimum in the orbital optimization landscape. In Super-CI, the stationarity condition, eq 10, is achieved by iteratively minimizing the overlaps between |REF⟩ and its singly excited configurations ​(the Brillouin states). The Super-CI wave function is written as

12 $$|SCI\rangle =\left(1+\sum_{p>q}s_{pq}\left({Ê}_{pq}-{Ê}_{qp}\right)\right)|REF\rangle$$

where the coefficients *s*_*pq*_ are determined from a secular equation. During the SCF optimization, the effects of the Brillouin states are iteratively folded into the reference, leading to vanishing *s*_*pq*_ at convergence. Further details can be found in refs 86–88 .

## 3 Computational Details

CSF-ROHF and SSG-CSF-ROHF calculations were performed using OpenMolcas.^61^ Single-CSF RDMs were evaluated externally using a Python interface^190^ that automatically supplies the RDMs to the RASSCF module of OpenMolcas, where the Super-CI orbital optimization was carried out. The Python interface is also part of the Tools of OpenMolcas (under the name CSF-ROHF_interface).^191^ SSG-RDMs were obtained with the SSG method,^68^ as implemented in the development version of the NECI codebase.^89,90^ Figure 2 illustrates the workflow of ​(SSG‑)​​​CSF-ROHF where ​(SSG‑)​​​RDMs are evaluated once at the beginning of the calculation and then fed into the orbital optimization loop.

**Figure 2:**
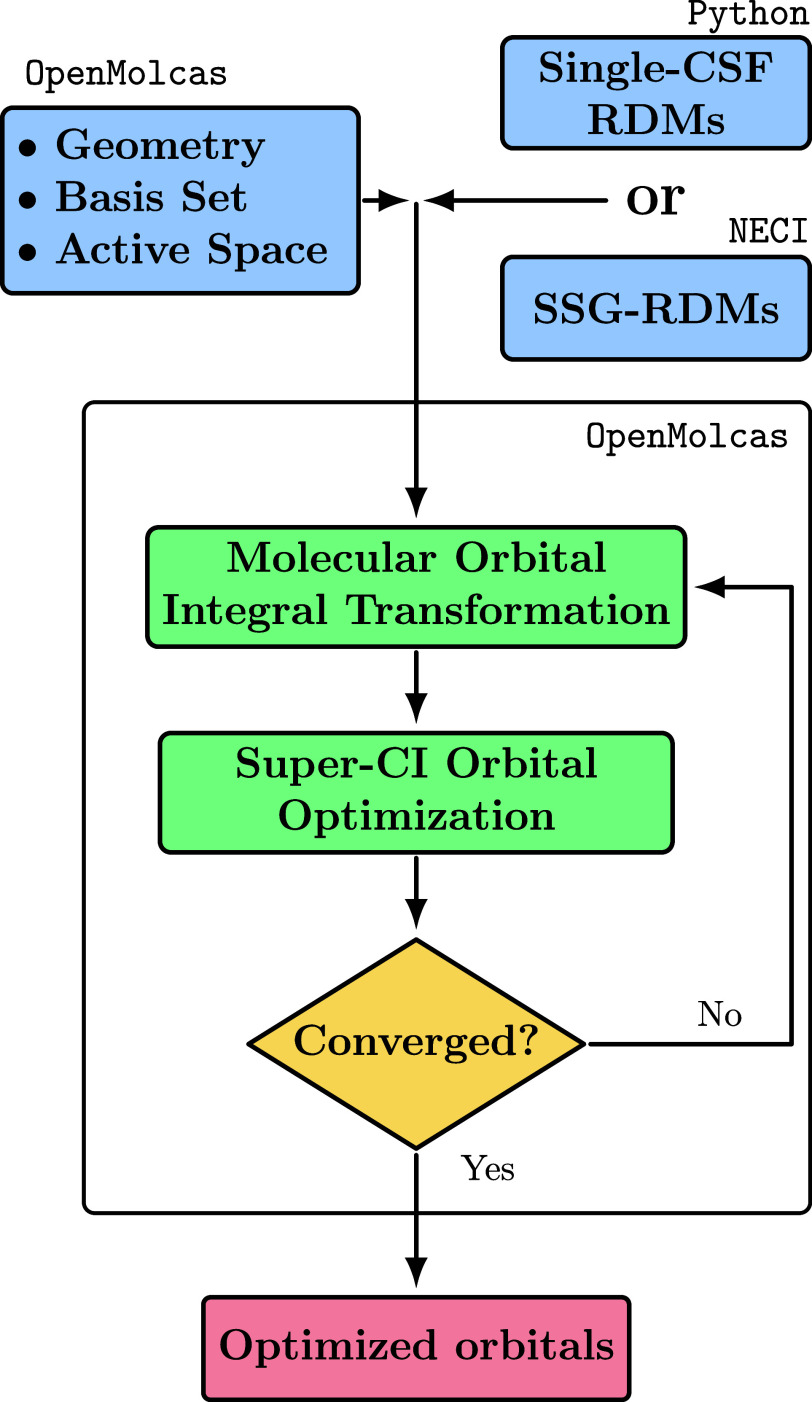
Workflow of ​(SSG‑)​​​CSF-ROHF. CSF-ROHF and SSG-CSF-ROHF use single-CSF RDMs and SSG-RDMs, respectively. The Python, NECI,^89,90^ and OpenMolcas^61^ text labels indicate the Python interface^190,191^ and the respective software used for each step.

The computational details for the benchmarks presented in Section 4 are summarized below.

First, in Section 4.1, we employed the STO-3G minimal basis set for the N_2_ calculations, allowing for detailed exploration of the optimization landscape. The atomic orbitals were rotated as illustrated in the section and used as the initial guess for the orbital optimization.

Second, for the performance tests in Section 4.2, the geometry of the largest of the {[Ni^II^​(H_2_O)_4_]_*n*_O_*n*–1_​(H_2_O)_2_}^2+^ chains ​(*n* = 10) was taken from ref 55, while the smaller chains ​(*n* = 1, ..., 9) were generated by removing fragments from the larger chain, while shifting the terminal water molecule to maintain the same distance to the nearest Ni atom. This strategy was adopted to keep the number of fragment optimizations at its bare minimum, while ensuring a rigorous assessment of the scaling of the method with respect to system size. In line with ref 55, a Def2-TZVP basis set with the Def2/J auxiliary basis for the resolution of the identity approximation were employed.^91,92^

Third, the geometries of [Fe­(III)_2_​S_2_​(SCH_3_)_4_]^2–^ and [Fe(III)_4_​S_4_​(SCH_3_)_4_] utilized in Section 4.3, were taken from our earlier works.^69,83^ For both clusters, the Fe atoms were described using the ANO-RCC-VDZ basis set, while all remaining atoms were described using the ANO-RCC-MB basis set.^93^ The orbital ordering employed for the [Fe​(III)_4_S_4_​(SCH_3_)_4_] calculations is discussed in Section 4.3.2. The neutral P-cluster geometry was taken from ref 3. The Fe atoms were described using the ANO-RCC-VDZP basis set, the S atoms using the ANO-RCC-VDZ basis set, and all remaining atoms using the ANO-RCC-MB basis set.^93^ All P-cluster calculations employed the orbital ordering ​(3–4–2–1–8–7–5–6) as reported in ref 94, with site labeling consistent with Figure 9 of that work. For [Fe​(III)_4_S_4_​(SCH_3_)_4_] and P-cluster calculations, the Cholesky-decomposition resolution-of-identity approximation^95–99^ was used to efficiently evaluate the electron repulsion integrals.

**Figure 3:**
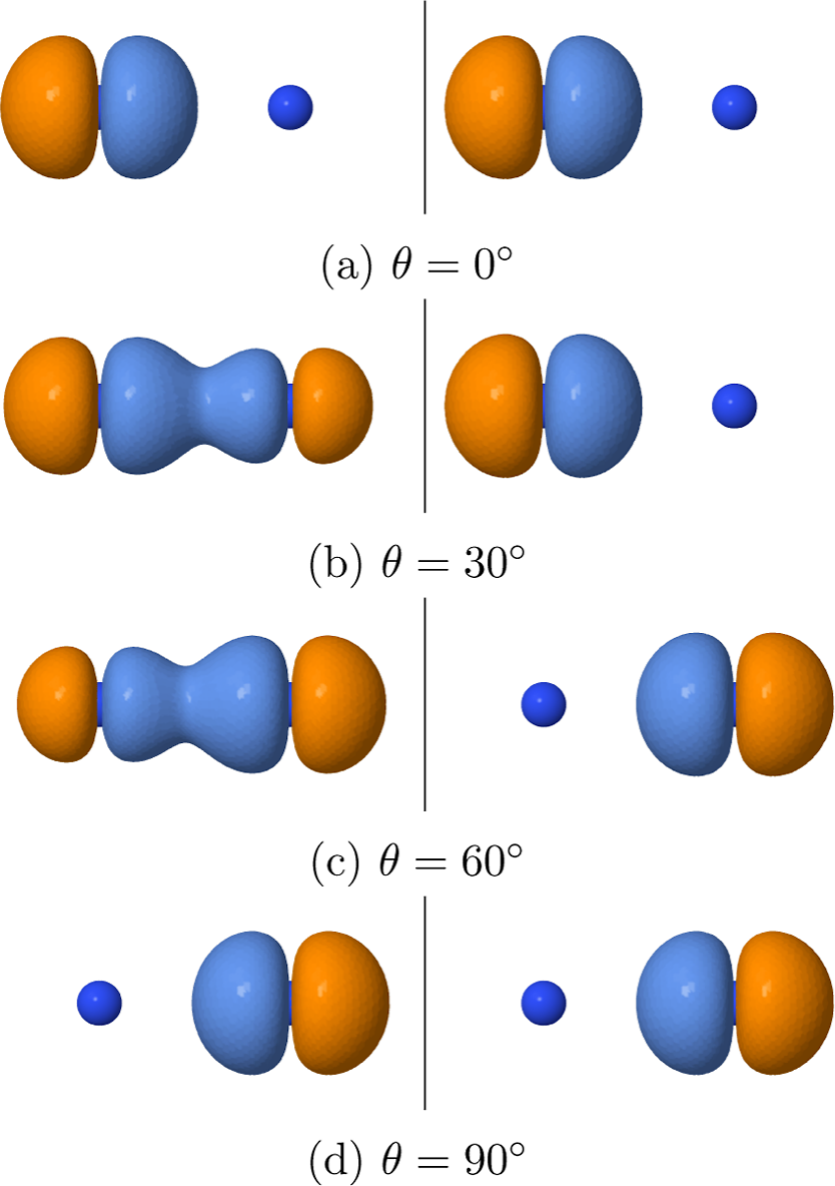
First 2p_*z*_ orbitals of N_2_ at *r* = 3.0 Å before ​(left) and after ​(right) CSF-ROHF orbital optimization, starting from different pairwise rotations of the 2p_*z*_ orbitals ​(indicated by the rotation angle θ). (a) θ = 0°, (b) θ = 30°, (c) θ = 60°, and (d) θ = 90°.

**Figure 4:**
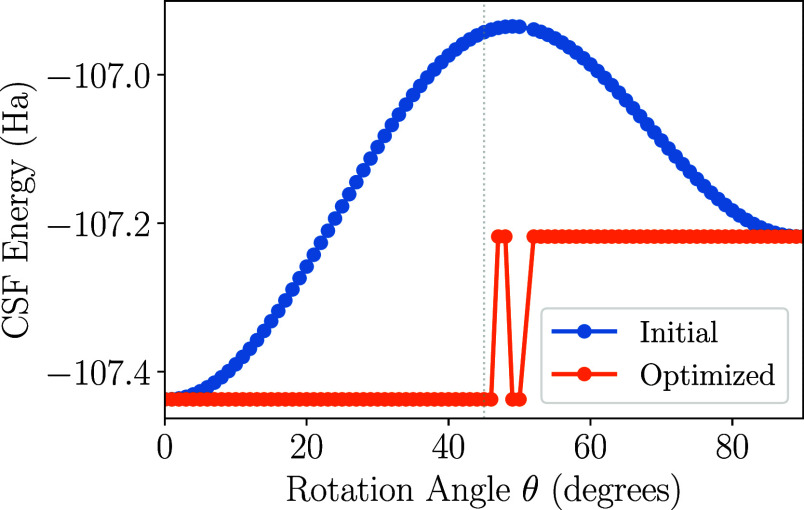
Target CSF energies before ​(blue) and after ​(orange) CSF-ROHF orbital optimizations. The rotation angle θ controls the pairwise mixing of the two 2p_*z*_ orbitals on each nitrogen atom.

**Figure 5:**
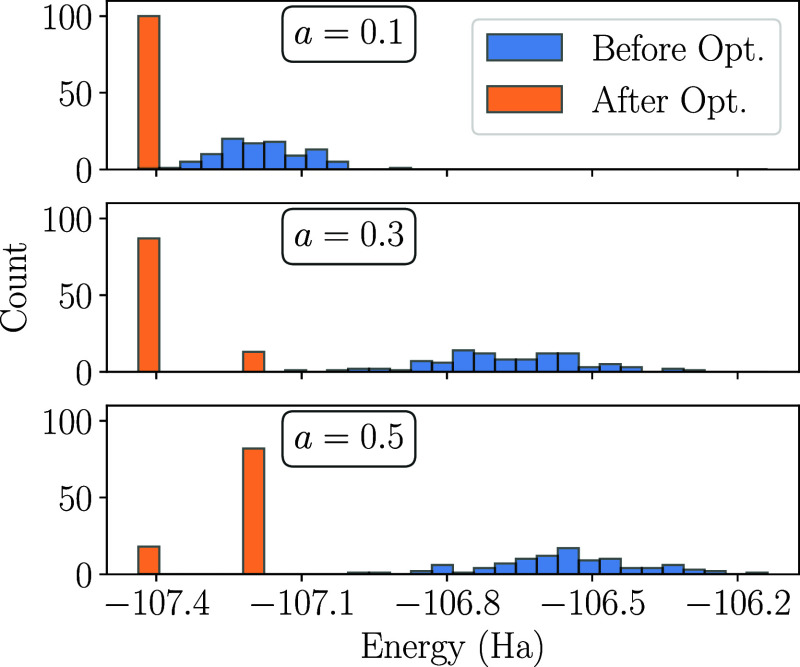
Histograms of CSF energies before ​(blue) and after ​(orange) CSF-ROHF orbital optimizations starting from randomly rotated 2p orbitals. The parameter *a* controls the amplitude of the random rotations. For each value of *a*, 100 independent sets of randomly rotated orbitals were used as starting orbitals for the optimizations.

**Figure 6:**
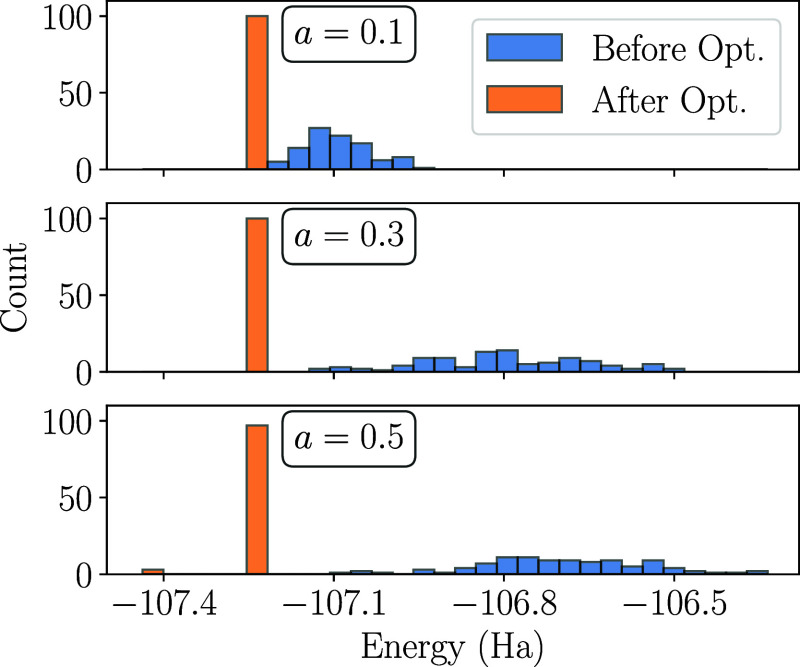
Histograms of CSF energies before ​(blue) and after ​(orange) CSF-ROHF orbital optimizations starting from orbitals obtained by randomly rotating a set of local-minimum 2p orbitals shown in Figure S1. The parameter *a* controls the amplitude of the random rotations.

**Figure 7:**
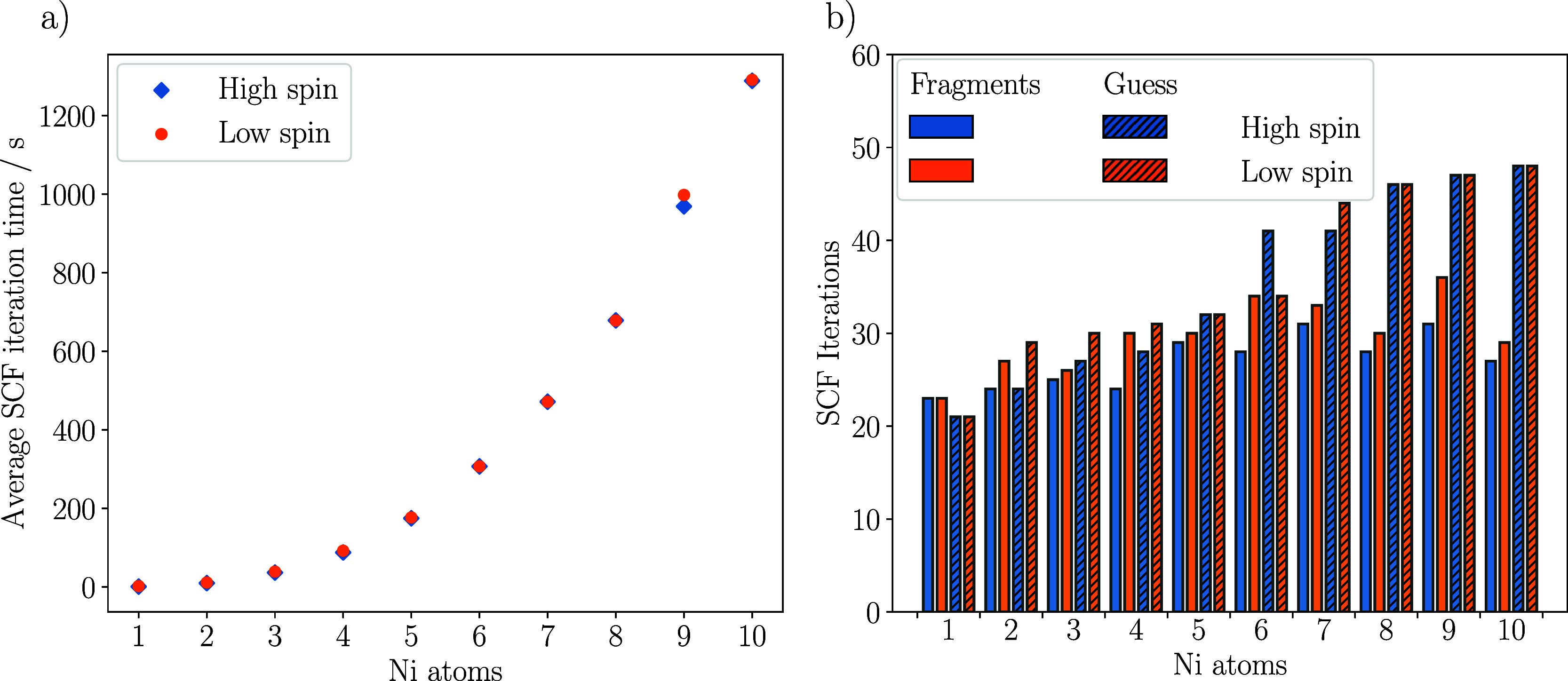
​(a) Average time per SCF iteration for HS and LS calculations as a function of the number of Ni atoms in the chain ​(*n*). ​(b) Number of SCF iterations needed to reach convergence, depending on *n*, spin, and type of initial orbitals.

**Figure 8:**
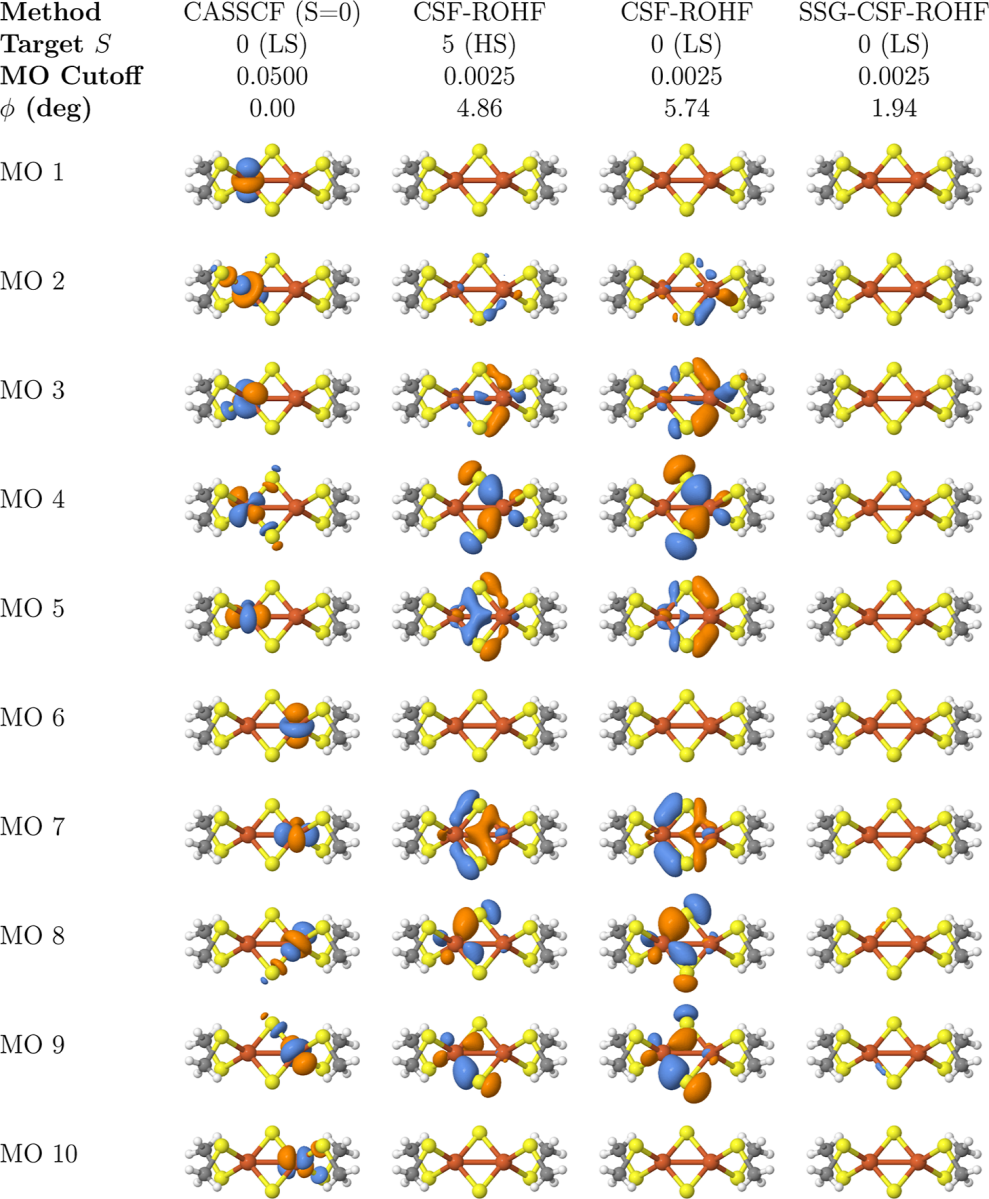
Localized CASSCF­(10,10) orbitals ​(left column) for the *S* = 0 state, and their differences with the HS-CSF-ROHF, LS-CSF-ROHF ​(*S* = 0), and SSG-CSF-ROHF ​(*S* = 0) orbitals ​(second, third and fourth columns, respectively). For the difference plots, the CASSCF orbitals are rotated to best match the compared orbital set, enabling MO-by-MO comparisons. A smaller isosurface cutoff value of 0.0025 is used to visualize the differences, as they are not visible with the cutoff ​(0.05) used for the CASSCF orbitals. The metric ϕ quantifies the similarity between the CASSCF orbital subspace and the compared orbital subspace.

**Figure 9:**
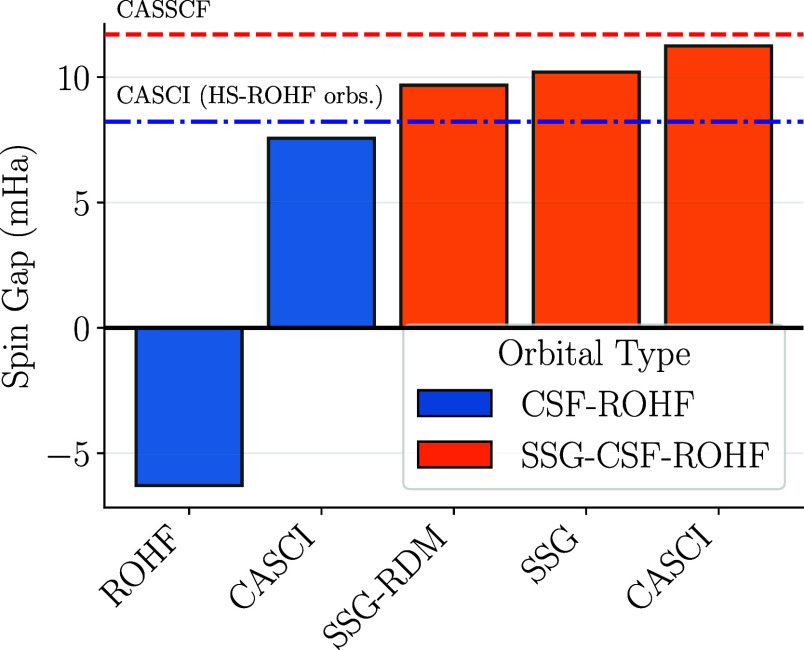
*E*​(*S* = 5) – *E*​(*S* = 0) spin gap of the [Fe​(III)_2_S_2_​(SCH_3_)_4_]^2–^, computed at various levels of theory. The bar colors indicate the type of orbitals used: blue for CSF-ROHF orbitals and orange for SSG-CSF-ROHF orbitals. The blue horizontal line denotes the CASCI spin gap obtained using HS-ROHF orbitals. The red horizontal line indicates the CASSCF reference value taken from ref 44.

The molecular geometries, representative input files for the CSF-ROHF and SSG calculations of N_2_, {[Ni^II^​(H_2_O)_4_]_*n*_O_*n*–1_​(H_2_O)_2_}^2+^, [Fe​(III)_2_S_2_​(SCH_3_)_4_]^2–^, [Fe​(III)_4_S_4_​(SCH_3_)_4_], and the P-cluster are provided in the Supporting Information.

All plots were generated using Matplotlib,^100^ and molecular visualizations were produced using Jmol.^101^

## 4 Results and Discussion

In this section, several aspects of CSF-ROHF and its perturbative extension, SSG-CSF-ROHF, are discussed. The convergence behavior of CSF-ROHF with respect to different initial orbital guesses is investigated in Section 4.1, where the results for the N_2_ molecule at an internuclear distance of 3 Å are presented. Here the focus is the role of intra-active orbital rotations.

The computational efficiency of CSF-ROHF, in terms of iteration count to convergence and wall time, is assessed in Section 4.2, where the results for the [Ni​(H_2_O)_4_]_n_O_n–1_​(H_2_O)_2_^2+^ ​(*n* = 1, ..., 10) model systems are presented, in which the system size is systematically increased.

The failure of CSF-ROHF and the subsequent improvements introduced by our SSG-CSF-ROHF are discussed in Section 4.3, where results for the challenging electronic structures of [Fe​(III)_2_S_2_​(SCH_3_)_4_]^2–^, [Fe​(III)_4_S_4_​(SCH_3_)_4_], and the nitrogenase P-cluster are presented and compared with previous results obtained at higher level of theory.^44,94^

### 4.1 Convergence of CSF-ROHF

For the N_2_ model system, the six 2p orbitals on the two nitrogen atoms were included in the active space—what we would normally refer to as a CAS​(6,6)—for which the |*uuu ddd*⟩ reference CSF can be defined. Two types of molecular orbital ​(MO) transformations were considered to investigate the convergence behavior of CSF-ROHF with respect to starting orbitals, with particular focus on the role of intra-active orbital rotations: ​(i) controlled rotations of the single 2p_*z*_ orbital-pair, and ​(ii) random active orbital rotations of all six 2p orbitals.

#### 4.1.1 Pairwise Rotations of the 2p_*z*_ Orbitals

The rotation angle θ was varied from 0° to 90°. The resulting rotated orbitals were used as initial guess for CSF-ROHF optimization. As θ increases from 0° ​(Figure 3a), the first 2p_*z*_ orbital, initially localized on the first nitrogen atom, gradually mixes with the 2p_*z*_ orbital on the second nitrogen atom ​(Figure 3b). At θ = 45°, both orbitals contain equal contributions from the two nitrogen atoms. Further increasing θ reverses the dominant character, such that the first 2p_*z*_ orbital becomes primarily localized on the second nitrogen atom and vice versa ​(Figure 3c). At θ = 90°, the resulting orbitals are equivalent to the original ones up to a swap in orbital ordering ​(Figure 3d).

The CSF-ROHF optimization yields fully localized orbitals ​(Figure 3, right panels) residing on the same nitrogen atoms on which the corresponding starting orbitals had dominant 2p_*z*_ character. Thus, two sets of orbitals are identified upon optimization, differing in the ordering of the two 2p_*z*_ orbitals. For θ ≲ 45°, the optimized orbitals consist of three 2p orbitals localized on the first nitrogen atom, followed by three 2p orbitals localized on the second nitrogen atom. Combined with the target CSF ​(|*uuu ddd*⟩), this implies that the first ​(last) three electrons reside on 2p orbitals on the first ​(second) site are coupled to a quartet, and the two quartets are antiferromagnetically coupled. On the contrary, for θ ≳ 50°, the optimized orbitals follow the 2p_*x*_^A^2p_*y*_^A^2p_*z*_^B^2p_*x*_^B^2p_*y*_^B^2p_*z*_^A^ ordering ​(note the swap in the ordering of the 2p_*z*_ orbitals). In this ordering, it is not possible to assign the first ​(or last) three electrons to the first ​(or second) nitrogen atom with local quartet spin coupling. The two groups of optimized orbitals correspond to two distinct stationary points in the orbital optimization landscape, with different CSF energies.

Figure 4 shows the target CSF ​(|*uuu* *ddd*⟩) energies before and after the CSF-ROHF orbital optimizations.

Optimizations starting from orbitals with θ ≲ 45°, where the first ​(second) admixed orbital is dominated by the 2p_*z*_ character on the first ​(second) nitrogen atom, converge to the same CSF-ROHF energy. In contrast, optimizations starting from orbitals with θ ≳ 50° converge to a stationary point with a higher energy. The orbital sets with 45 ≲θ≲ 50° appear to be near the boundary between the two groups and converge to either of the two CSF energies.

Interestingly, the optimized CSF energy of the second group is identical to that of the first group upon swapping the ordering of the two 2p_*z*_ orbitals, suggesting that the two stationary points are related by a symmetry operation corresponding to the swap of the two 2p_*z*_ orbitals. But the resulting single-CSF wave function is not invariant under this operation, leading to two distinct stationary points in the orbital optimization landscape.

#### 4.1.2 Random Rotations of 2p Orbitals

Next, we examine the convergence behavior of the CSF-ROHF method starting from randomly rotated orbitals of the nitrogen molecule. The random rotations are only applied to the six active 2p orbitals. The extent of the random rotations is controlled by a parameter *a*, with larger values corresponding to more significant deviations from the reference orbitals. Details of the random rotation procedure are provided in Appendix A.

Figure 5 shows histograms of the CSF energies before and after CSF-ROHF orbital optimizations for different values of *a*.

After optimization, two distinct CSF energies are observed, corresponding to the two energies already seen in Figure 4. Larger random rotations ​(higher *a* values) increase the likelihood of the orbitals converging to the higher-energy local minimum. Random rotations that significantly mix the 2p orbitals can be related to θ ≳ 50° in the pairwise rotation analysis and they are more likely to converge to the higher-energy local minimum. On the other hand, random rotations that preserve the dominant character of the original site-separated orbitals are more likely to converge to the lower-energy local minimum.

We then selected one set of optimized orbitals that had converged to the higher energy local minimum ​(Figure S1), applied random rotations to them, and repeated the CSF-ROHF orbital optimizations. As shown in Figure 6, a larger fraction of optimizations became trapped in the local minimum compared to the previous test. We observed that some orbital orderings allow CSF-ROHF orbital optimization to escape higher-energy local minima, showing that orbital ordering is crucial for convergence. These results emphasize the importance of a well-chosen initial guess to reach the desired minimum. Our genetic-algorithm-driven Quantum Anamorphosis strategy offers a potentially useful approach to address this issue.^94^

### 4.2 Scaling of CSF-ROHF: the {[Ni​(H_2_O)_4_]_n_O_n–1_​(H_2_O)_2_}^2+^ Stress Test

We assess the performance of our CSF-ROHF implementation by comparing a series of calculations on linear clusters of the form {[Ni^II^​(H_2_O)_4_]_*n*_O_*n*–1_​(H_2_O)_2_}^2+^ ​(*n* from 1 to 10), similar to what was done in ref 55. Each Ni atom in the chain contributes two triplet-coupled unpaired electrons. We performed both HS calculations ​(where *S* = *n*) with the standard CASSCF implementation of OpenMolcas and LS calculations ​(where *S* = 0 for even *n* and *S* = 1 for odd *n*) with CSF-ROHF. Additionally, we used two different strategies for generating the initial orbitals. In one case ​(“fragments”) we performed individual calculations for the different fragments [Ni​^II^(H_2_O)_4_]^2+^, O^2–^, terminal H_2_O) and simply juxtaposed the fragment orbitals. For the [Ni​^II^(H_2_O)_4_]^2+^ we did CASSCF​(2,2) calculations, with *S* = 1, for the other fragments they were simple restricted Hartree–Fock calculations. In the other case ​(“guess”) we took the default guess orbitals generated by OpenMolcas ​(see ref 102 for a description). The active orbitals are the two e_g_-like orbitals of each Ni center, ordered in their “natural” order along the chain: first the two orbitals of a terminal Ni, then the two orbitals of the closest Ni atom, etc. With this order, the CSF targeted in the CSF-ROHF calculations was |*uu* *dd* *uu* *dd*...⟩ for all chain lengths.

The time per SCF iteration and the number of iterations required to reach convergence are shown in Figure 7. In the left panel we observe the expected superlinear increase in computational time as the basis set size increases ​(from 303 basis functions with *n* = 1 to 2535 with *n* = 10). Importantly, the timings are essentially the same for HS and LS calculations, indicating that the CSF-ROHF treatment does not introduce any significant overhead. In the right panel we see that all calculations converge in 20 to 50 iterations. Typically LS requires a few more iterations than HS, but not to the extent of making the calculation much more costly. The type of initial orbitals is found to have a more significant influence on the iteration count, especially in longer chains, with the “fragments” orbitals being clearly advantageous, always converging in under 40 iterations.

Overall, the convergence performance and scaling of LS-CSF-ROHF is very similar to HS-CSF-ROHF, as in both cases the CI expansion is limited to a single CSF. We refrain from making direct timing comparisons with ref 55, as the hardware, parallel environment, integral screening defaults, etc. are different in the two implementations. We note, however, that our calculations were performed on an Intel Core i9-14900K processor, using 4 GB of RAM and a single process, but with multithreaded ​(8 threads) linear algebra libraries.

### 4.3 Real World Chemistry: Where Dynamic Correlation Matters

This section is devoted to the application of ​(SSG‑)​CSF-ROHF to the [Fe​(III)_2_S_2_​(SCH_3_)_4_]^2–^, [Fe​(III)_4_S_4_​(SCH_3_)_4_], and P-cluster. In line with the scope of this work, we applied our protocol to all collinear singlet states of the iron–sulfur clusters.

First, we show that the simple CSF-ROHF method, fails in two ways: ​(a) it is not superior to HS-ROHF in providing good initial orbitals for subsequent MC-SCF calculations targeting LS states of large polynuclear transition metal clusters, and crucially ​(b) in absence of dynamic correlation, it provides qualitatively incorrect description of the spin gaps in these clusters. Second, we demonstrate that the SSG-CSF-ROHF method can overcome these limitations and provides spin-gap predictions in excellent agreement with high-level multireference calculations at the modest near-mean-field cost of ROHF.

#### 4.3.1 [Fe​(III)_2_S_2_​(SCH_3_)_4_]^2–^

We will compare the CSF-ROHF and SSG-CSF-ROHF methods for the [Fe​(III)_2_S_2_​(SCH_3_)_4_]^2–^ cluster, which is the smallest prototypical system for studying exchange interactions across the magnetic sites in polynuclear transition metal clusters based on the [FeS] motif.

#### 4.3.2 Comparison of Low-Spin and High-Spin CSF-ROHF Orbitals

The ten Fe​(III) 3d magnetic orbitals were considered as active. HS-ROHF orbitals were used as initial guess for the CSF-ROHF optimization after localization and site-reordering ​(Figure 8, first column). The |*uuuuu ddddd*⟩ CSF was used to drive the LS-CSF-ROHF optimization.

As shown in Table 3, the HS-CSF-ROHF energy is lower than the LS-CSF-ROHF energy, and the resulting spin gap ​(*E*​(*S* = 5) – *E*​(*S* = 0) = – 6.29 mHa) is qualitatively incorrect as compared to the 11.7 mHa value obtained at the CASSCF level of theory.^44^ Similar qualitative failures of CSF-ROHF spin gaps for the [Fe​(III)_2_S_2_​(SCH_3_)_4_]^2–^ have been reported in previous CSF-ROHF studies.^55,56^

**Table 3:** Energies ​(in Ha) Obtained From CSF-ROHF, CASCI​(10,10), SSG-RDM, and SSG Using CSF-ROHF and SSG-CSF-ROHF Orbitals Optimized for LS- and HS-States of the [Fe​(III)_2_S_2_​(SCH_3_)_4_]^2–^ ^a^ ^b^

		LS-optimized orbitals		HS-optimized orbitals	
orbital type	method	*S* = 0	*S* = 5	*S* = 0	*S* = 5
CSF-ROHF	CSF-ROHF	–0.716340	–	–	–0.722630
CSF-ROHF	CASCI	–0.730192	–0.721821	–0.730851	–0.722630
SSG-CSF-ROHF	SSG-RDM	–0.732308	–	–	–0.722630
SSG-CSF-ROHF	SSG	–0.732831	–0.720671	–0.730273	–0.722630
SSG-CSF-ROHF	CASCI	–0.733879	–0.720673	–0.730851	–0.722630
CASSCF	CASSCF	–0.73433219			

a Shifted by 5092 Ha.

b The LS-optimized orbitals were obtained via ​(SSG‑)​​CSF-ROHF targeting the |*uuuuu ddddd*⟩ CSF. The CASSCF singlet energy is provided for reference.

To assess the quality of the two orbital sets in post-HF methods, we performed CASCI​(10,10) calculations using both LS- and HS-optimized ROHF orbitals. As shown in Table 3 ​(Orbital Type = CSF-ROHF), the HS-optimized orbitals yield lower CASCI energies for both the LS and HS states than the LS-optimized orbitals. This indicates that, within CASCI, the HS-optimized orbitals provide a variationally more accurate description of both spin states—and potentially closer to convergence in CASSCF optimizations—and that CSF-ROHF orbital optimization does not confer a clear advantage over HS-ROHF orbitals, even for post-HF calculations targeting the LS state.

#### 4.3.3 Incorporating Dynamic Correlation Based on Löwdin Downfolding

To incorporate correlation effects which are present in the CAS​(10,10) space, but absent in the CSF-ROHF method, we performed SSG calculations in which the P space contains only the target CSF ​(|*uuuuu ddddd*⟩), while the Q space contains all other CSFs within the same CAS​(10,10) space—mostly metal-to-metal CT and non-Hund configurations. Note that the SSG correction does not change the HS state energy of the CAS​(10,10), as |*uuuuu uuuuu*⟩ is already the exact wave function of the HS state within this space. Because the P space consists of a single CSF, the SSG calculation converges very rapidly ​(approximately 30 s with a single CPU core), indicating that this step introduces negligible computational overhead.

Table 3 also summarizes the energies obtained with various methods using the SSG-CSF-ROHF orbitals. Notably, in contrast to the CSF-ROHF results, SSG-CSF-ROHF yields lower energies for the LS state ​(*S* = 0) with LS-optimized orbitals than with HS-optimized orbitals. This demonstrates the rationale for performing orbital optimization for the LS state rather than relying on conventional HS-ROHF, which is not apparent from the CSF-ROHF results alone.

Despite the fixed-RDM approximation, SSG-CSF-ROHF yields a qualitatively correct spin gap ​(SSG-RDM, 9.68 mHa). Performing a subsequent SSG calculation using the optimized orbitals ​(therein removing the fixed-RDM approximation) leads to a further improvement of the spin gap ​(SSG, 10.2 mHa), in excellent agreement with the CASSCF reference value of 11.7 mHa.^44^ Although CASCI on the SSG-CSF-ROHF orbitals provides the most accurate spin gap, the gain is not significant enough to justify the additional computational cost. Noticeably, the SSG-CSF-ROHF spin gap is already in better agreement with the CASSCF reference than the CASCI spin gap obtained with the HS-optimized orbitals, which are commonly used as initial guess for CASSCF optimizations.

The quality of the three orbital sets, namely HS-CSF-ROHF-optimized, LS-CSF-ROHF-optimized, and LS-SSG-CSF-ROHF-optimized orbitals, are assessed by comparing them with CASSCF​(10,10) orbitals ​(Figure 8).

When visualized using the isosurface cutoff ​(0.05), all orbital sets, including the CASSCF orbitals, exhibit clear Fe 3d character and the orbital sets are indistinguishable by visual inspection. To resolve differences among the three ROHF-based orbital sets, we therefore visualize the differences between the ROHF orbitals and the rotated CASSCF orbitals with a smaller isosurface cutoff ​(0.0025). For each comparison, the CASSCF orbitals are rotated to maximally match the target orbital set using an energy-invariant Procrustes transformation,^103,104^ enabling a meaningful MO-by-MO comparison.

Figure 8 also reports the metric $\phi =\arccos\left(\prod_{i=1}^{10}\;\cos\theta_{i}\right)$ , where θ_*i*_ is the *i*th principal angle between the target set of orbitals and the CASSCF orbitals.^105^ Numerically, cos θ_*i*_ are the singular values of the matrix product **C**′**SC**, where **C**′ and **C** are the MO coefficient matrices of the CASSCF and target orbitals, respectively, and **S** is the atomic orbital overlap matrix. ϕ represents the angle between the two Euclidean subspaces spanned by the active orbitals of the CASSCF and target orbital spaces, respectively, with smaller values indicating greater similarity.^106^ Both the orbital difference and the ϕ values indicate that the SSG-CSF-ROHF orbitals are the closest to the CASSCF orbitals among the three sets. This observation rationalizes the energy results, as the SSG-CSF-ROHF calculations yield CASCI energies closest to the CASSCF reference values ​(Table 3).

The lowest-to-highest spin gap of the [Fe​(III)_2_S_2_​(SCH_3_)_4_]^2–^, computed using the approaches described above, are summarized in Figure 9, together with the CASSCF reference value ​(red horizontal line) and the CASCI spin gap obtained using HS-ROHF orbitals ​(blue horizontal line). The blue horizontal line represents a strategy typically utilized when CASSCF calculations are too expensive.^3,69,83,94^

These results highlight the importance of incorporating dynamic correlation effects into the orbital optimization procedure beyond the CSF-ROHF approach to accurately describe the spin states of strongly correlated systems such as the [Fe​(III)_2_S_2_​(SCH_3_)_4_]^2–^. Performing a CASCI calculation using the SSG-CSF-ROHF orbitals yields a spin gap which is indistinguishable in practice to the CASSCF reference value. The computational cost of the SSG-CSF-ROHF approach is only slightly higher than that of CSF-ROHF, as the SSG calculations involve only a single CSF in the P space, and the computationally demanding step ​(the CASCI) is performed only once, at the end of the orbital optimization procedure. Even the results obtained at the SSG-CSF-ROHF level ​(labeled SSG in Figure 9), which are obtained without performing a more costly CASCI calculation, yield a spin gap which is improved as compared to the CASCI often used in the literature to avoid the more expensive CASSCF. These results make the present approach particularly attractive for large systems where full CAS treatments become prohibitively expensive.

#### 4.3.4 [Fe​(III)_4_S_4_​(SCH_3_)_4_]

The increased number of magnetic centers in the [Fe​(III)_4_S_4_​(SCH_3_)_4_] cluster compared to the [Fe​(III)_2_S_2_​(SCH_3_)_4_]^2–^ introduces additional complexity due to the nontrivial site ​(orbital) ordering effects, that go beyond the simple two-site separation, and the possibility to target different collinear electronic states, namely the |*UUDD*⟩ and |*UDUD*⟩ states, where *U* ​(*D*) denotes a site with *uuuuu* spin-up ​(*ddddd* spin-down) alignments. We adopt the optimal orbital ordering proposed in ref 69, which allows to achieve a highly compact representation of the low-energy states, and selective targeting of specific spin states. As shown in Figure 5 of ref 69, the |*UUDD*⟩ and |*UDUD*⟩ states are the lowest and highest singlet states, respectively, of the [Fe​(III)_4_S_4_​(SCH_3_)_4_] cluster investigated in this work. They are separated by a small energy gap ​(3.2 mHa), and their wave functions are maximally compressed within the Quantum Anamorphosis strategy. For these two collinear singlet states, we carry out the same analysis as for the [Fe​(III)_2_S_2_​(SCH_3_)_4_]^2–^ cluster.

Table 4 summarizes the energies obtained from CSF-ROHF orbital optimizations targeting the |*UUDD*⟩, |*UDUD*⟩, and |*UUUU*⟩ states, together with the corresponding stochastic CASCI​(20,20) reference energies.

**Table 4:** CSF-ROHF, SSG-RDM, SSG, and Stochastic CASCI​(20,20) Energies^a^ ​(in Ha) for the [Fe​(III)_4_S_4_​(SCH_3_)_4_] Cluster, Obtained Using ​(SSG‑)​​CSF-ROHF |*UUDD*⟩, |*UDUD*⟩, and |*UUUU*⟩ Orbitals^e^

	UUDD-opt.		UDUD-opt.		UUUU-opt.	
method	*S* = 0	*S* = 10	*S* = 0	*S* = 10	*S* = 0	*S* = 10
CSF-ROHF^b^	–0.510982	–	–0.512516	–	–	–0.524527
CASCI^b^	–0.537576	–0.522761	–0.534502	–0.523315	–0.538431	–0.524527
SSG-RDM^c^	–0.540929	–	–0.535090	–	–	–0.524527
SSG^c^	–0.541801	–	–0.535559	–	–	–0.524527
CASCI^c^	–0.544314	–	–0.538459	–	–	–0.524527
CASSCF^d^	–0.545174	–	–	–	–	–0.524527

a Shifted by 8432 Ha.

b CSF-ROHF orbitals.

c SSG-CSF-ROHF orbitals.

d CASSCF orbitals.

e The *S* = 0 calculations for the UDUD-optimized orbitals target the |*UDUD*⟩ state, while the other *S* = 0 calculations target the |*UUDD*⟩ state. The CASSCF​(20,20) energy of the |*UUDD*⟩ state is taken from ref 44.

Consistent with the results for the [Fe​(III)_2_S_2_​(SCH_3_)_4_]^2–^, the ordering of the CSF-ROHF energies is opposite to that of the CASCI​(20,20) energies, and the CASCI singlet energies computed using the *UUUU* ROHF orbitals are lower than those obtained from CSF-ROHF optimizations for the collinear singlet states. These results confirm the limitations of CSF-ROHF when describing LS states.

Next, we applied SSG-CSF-ROHF calculations with P={|UUDD⟩} or {|*UDUD*⟩} ​(see Table 4, rows marked with a *c* footnote). Incorporating dynamic correlation through SSG-CSF-ROHF reverses the ordering of the singlet energies relative to the CSF-ROHF results already at the lowest level of approximation ​(SSG-RDM). Subsequent SSG and CASCI​(20,20) calculations using the SSG-CSF-ROHF orbitals further lower the energies for both collinear singlet states.

As shown in Figure 10, spin gaps computed using SSG-CSF-ROHF orbitals are in good agreement with the CASSCF reference value of the |*UUDD*⟩ state. Notably, the SSG-CSF-ROHF results ​(the “SSG-RDM” bar) already provide similar or even better spin gaps than the corresponding CASCI calculations based on HS-ROHF orbitals ​(blue horizontal line), and further improvements are introduced by SSG or stochastic CASCI calculations that rely on the SSG-CSF-ROHF optimized orbitals. The CASCI​(20,20) spin gaps obtained with SSG-CSF-ROHF optimized orbitals show excellent agreement with the CASSCF reference, indicating that the present low-cost orbital optimization strategy yields CASSCF-quality orbitals for a minimal active space comprising only the Fe 3d orbitals.

**Figure 10:**
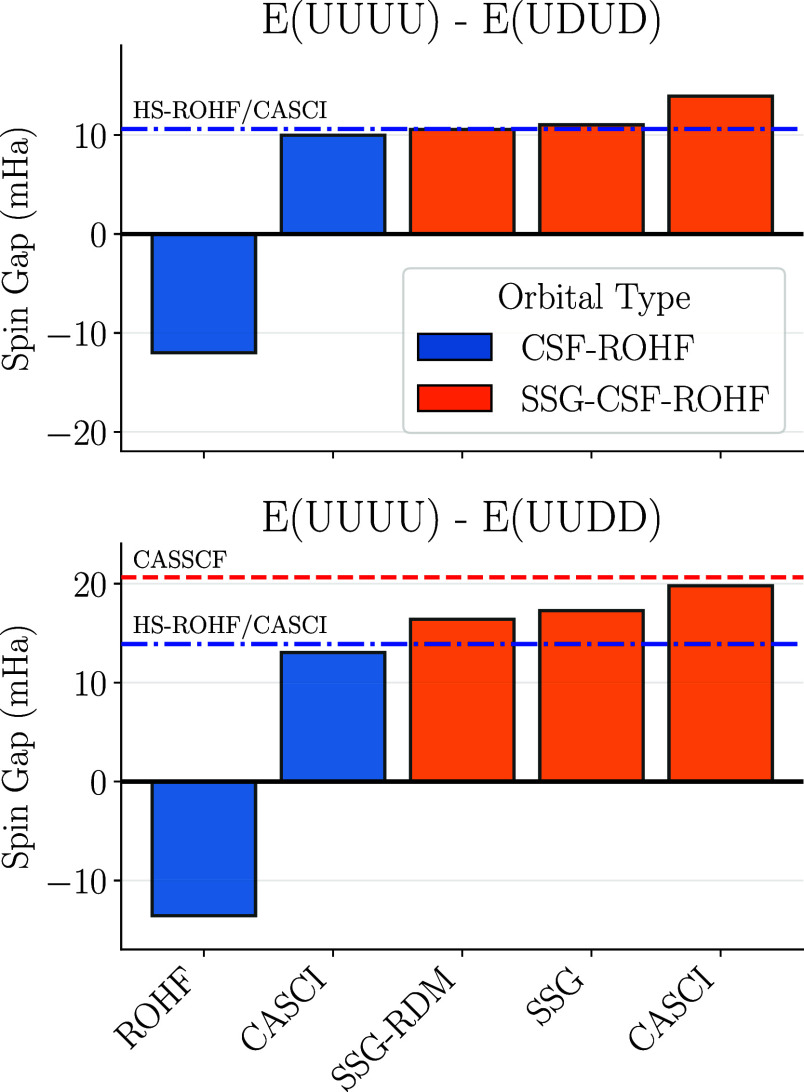
Spin gaps of the [Fe​(III)_4_S_4_​(SCH_3_)_4_] cluster computed at various levels of theory. The upper and lower plots show the *E*​(|*UUUU*⟩) – *E*​(|*UDUD*⟩) and the *E*​(|*UUUU*⟩) – *E*​(|*UUDD*⟩) spin gaps, respectively. Blue bars correspond to results obtained using CSF-ROHF orbitals, whereas orange bars indicate results obtained using the SSG-CSF-ROHF optimized orbitals. The blue horizontal line denotes the spin gap obtained from CASCI calculations using HS-ROHF orbitals.^69^ The red horizontal line indicates the CASSCF reference spin gap for the |*UUDD*⟩ state.^44^

#### 4.3.5 Nitrogenase P-Cluster

The observations for [Fe​(III)_2_S_2_​(SCH_3_)_4_]^2–^ and [Fe​(III)_4_S_4_​(SCH_3_)_4_]—that inexpensive orbital optimization can yield CASSCF-quality orbitals and energetics for minimal Fe 3d active spaces—motivate the application of the same paradigm to the larger CAS​(48,40) active space of a nitrogenase P-cluster model. We used the localized and site-ordered HS-ROHF orbitals from ref 94 as a starting guess, where each Fe​(II) center has one doubly occupied and four singly occupied 3d orbitals.

Figure 11 summarizes the relative energies obtained using several approaches of increasing accuracy, and three different sets of orbitals. Results with blue, green and red backgrounds correspond to calculations using optimized CSF-ROHF, SSG-CSF-ROHF, and the conventional HS-ROHF optimized orbitals, respectively.

**Figure 11:**
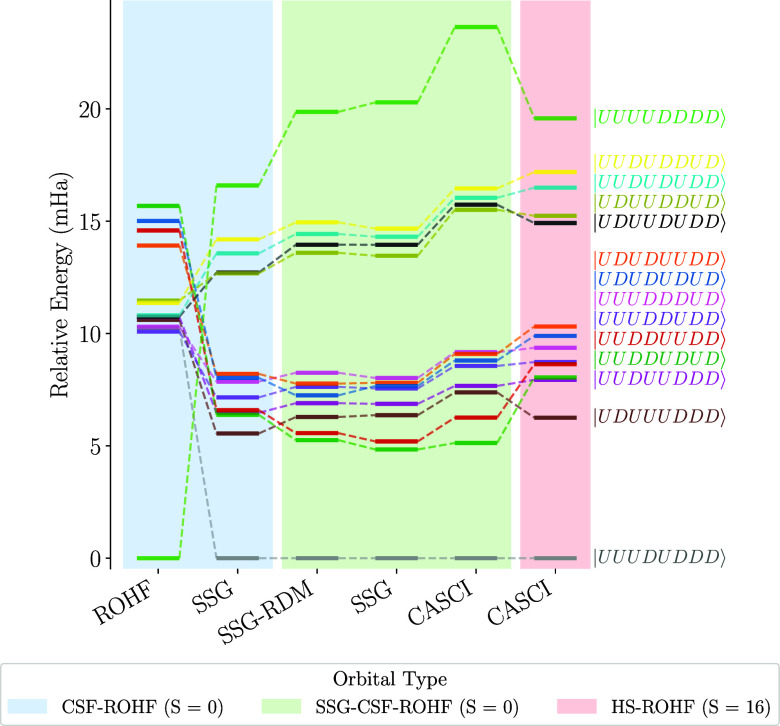
Energy levels of the 14 collinear singlet states of the nitrogenase P-cluster model, computed using various methods and orbital sets. The background color indicates the type of orbitals used, as denoted in the legend. CASCI​(48,40) energies using HS-optimized ROHF orbitals are taken from ref 94. RDM indicates energies evaluated from SSG-CSF-ROHF.

CSF-ROHF optimizations ​(blue background) were performed for all 14 low-energy collinear CSFs in a state-specific fashion ​(different orbital optimizations for each CSF). A SSG on the CSF-ROHF orbitals accounts for correlation effects beyond the single reference CSF, while missing the orbital response to these correlation effects. Following the strategy employed for the smaller clusters, state-specific SSG-CSF-ROHF orbital relaxations were performed, aiming at improved orbitals and energies ​(green background). The energies obtained from the SSG-CSF-ROHF while keeping the SSG-RDMs fixed are referred to as “SSG-RDM”. The energies obtained from SSG using the SSG-CSF-ROHF optimized orbitals are referred to as “SSG” in the same green background. The full stochastic CASCI​(48,40) calculation using the SSG-CSF-ROHF optimized orbitals is referred to as “CASCI” in the Figure 1.

Consistent with the [Fe​(III)_2_S_2_​(SCH_3_)_4_]^2–^ and [Fe​(III)_4_S_4_​(SCH_3_)_4_] results, CSF-ROHF energies ​(“ROHF” in blue background) produce qualitatively different energy orderings compared to the more accurate calculations, and they are not to be trusted.

All the remaining results show consistent patterns, where the 14 states energetically separate into four groups: ​(1) the |*UUUD UDDD*⟩ ground state, ​(2) a group of eight states, ​(3) a group of four states, and ​(4) the highest |*UUUU DDDD*⟩ state. Across all methods, while the states within each group are identical, the energy gaps between the groups and the relative stability of states within the same group may differ. These results demonstrate that incorporating dynamic correlation via SSG-CSF-ROHF in the orbital optimization yields CASCI-quality results at near-mean-field cost, avoiding the need for expensive Stochastic-CASCI or even harder Stochastic-CASSCF calculations.^41^

Assuming that the predictions for the P-cluster are similar to those for the smaller clusters, it is to be concluded that the best results are those obtained with the SSG-CSF-ROHF optimized orbitals ​(green background), followed by a single CASCI​(48,48) calculation. If we compare these results with the CASCI​(48,40) results obtained with the conventional HS-ROHF orbitals ​(red background), we can see that the SSG-CSF-ROHF optimized orbitals yield an overall larger lowest-to-highest spin gap ​(by about 5 mHa), and a minor different ordering of the states within the second group. For example, the second-lowest SSG-CSF-ROHF/CASCI state is |*UUDDUDUD*⟩, whereas it is |*UDUUUDDD*⟩ when using the HS-ROHF orbitals.

Notably, although both SSG and CASCI employ stochastic population dynamics in imaginary time, the computational cost of SSG calculations is markedly lower. This is due to the smaller effective Hilbert space and reduced stochastic noise, as well as the more stable walker dynamics observed in SSG simulations. Consequently, SSG calculations require far fewer walkers than the corresponding FCIQMC simulations, enabling efficient evaluation of large systems ​(see Figure S2).

## 5 Conclusions and Outlook

We have presented a new GUGA-based spin-adapted CSF-ROHF algorithm, that enables orbital optimization at mean-field cost for arbitrary spin states. Nonredundant orbital rotations between orbital subspaces are controlled by the GAS scheme, available in the OpenMolcas software, while the Super-CI method is employed to variationally minimize the energy. This integrative framework enables a straightforward and robust algorithm.

The convergence behavior of CSF-ROHF was investigated for the N_2_ molecule at an internuclear separation of *r* = 3.0 Å, demonstrating the critical role of the ordering of the starting orbitals. In particular, we showed that CSF-ROHF can escape local minima when an appropriate orbital ordering is employed. If orbital ordering of the starting orbitals is not optimal, the CSF-ROHF procedure can easily get trapped in local minima, with physical features that resemble those of excited states, such as non-Hund states.

Previous developments on CSF-ROHF methods, for which dynamic correlation effects beyond the single-CSF level are not included, have reported qualitatively incorrect relative stability of low-energy states in exchange-coupled systems, such as the small [Fe​(III)_2_S_2_​(SCH_3_)_4_]^2–^ cluster. We observe the same behavior in our CSF-ROHF calculations for [Fe​(III)_2_S_2_​(SCH_3_)_4_]^2–^, [Fe​(III)_4_S_4_​(SCH_3_)_4_], and P-cluster, where the spin gaps predicted at the CSF-ROHF level are inverted relative to those obtained from high level CASCI or CASSCF optimizations.

Importantly, we observed that the CSF-ROHF approach, when used as a variational orbital optimizer for subsequent post-HF calculations, does not offer any advantage over conventional HS-ROHF even though it allows one to target LS states directly. We rationalize this observation by noting that, for collinear spin states—which are the focus of this work—the dominant exchange interactions are already captured at the single-CSF level, while ionic contributions ​(through-space CT excitations in a localized orbital picture) are largely absent. These ionic contributions are essential for accurately describing the relative stability of LS states, and they are not present in CSF-ROHF methods. Although these missing CT effects are recovered by approaching the CAS limit, they can also be incorporated effectively through perturbative treatments. This observation motivates the inclusion of dynamic correlation at the orbital-optimization level without resorting to computationally demanding explicit multiconfigurational wave functions.

We extended CSF-ROHF to incorporate dynamic correlation effects based on Löwdin downfolding using the recently developed SSG method. The resulting SSG-CSF-ROHF strategy optimizes orbitals under the influence of dynamic correlation. Applications to [Fe­(III)_2_​S_2_​(SCH_3_)_4_]^2–^ and [Fe(III)_4_​S_4_​(SCH_3_)_4_] clearly reveal the advantage of the method over the simple CSF-ROHF approach. The SSG-CSF-ROHF approach yields CASSCF-quality orbitals and energetics for minimal active-space models of iron–sulfur clusters at near-mean-field cost. In pushing the current limits, we applied this approach to a nitrogenase P-cluster model with an active space of CAS​(48,40), reporting singlet energies for 14 collinear spin configurations and showing CASCI-quality energy orderings without performing expensive CI calculations.

Several avenues exist for the further development of the CSF-ROHF algorithm and its SSG-CSF-ROHF perturbative extension. First, although we have not observed a strong dependence of the CSF-ROHF procedure on the orbital ordering, provided that a site-separated ordering is employed ​(i.e., orbitals associated with the same magnetic center are adjacent), the use of an optimal ordering certainly remains beneficial at the SSG-CSF-ROHF level. In fact, a more compact wave function can improve the accuracy of the perturbative treatment ​(reduced error in the diagonal approximation in the QQ space).

In addition, the present protocol provides orbitals—and, via a subsequent SSG calculation, corresponding RDMs—that can be directly used to evaluate properties of collinear states beyond energies. It can also be generalized to state-averaged orbital optimization, in which RDMs are constructed as weighted averages over multiple targeted states, either within the CSF-ROHF or the SSG-CSF-ROHF methods.

While the CSF-ROHF discussed so far is exclusively tailored toward collinear states that are readily described by single GUGA CSFs, we can also envision a generalization that extends its applicability to noncollinear states. For a noncollinear CSF-ROHF algorithm, one constructs and optimizes a P space containing all necessary GUGA CSFs to describe the noncollinear state, and uses the corresponding RDMs to perform orbital optimization. Alternatively, a SplitGAS approach can be adopted, where the P is dressed by all couplings from Q space, which now accounts for hopping configurations from all references CSFs in P . The resulting RDMs are used to optimize the orbitals. These aspects will be the subject of future work.

## Supplementary Material





## A Appendix

### Random Orbital Rotation Scheme

We seek to construct unitary transformations that randomly rotate orbitals with a controllable amplitude

13
$$C^{\prime }=CU$$

where **C** and **C**′ are the original and transformed MO coefficient matrices, respectively, and **U** ^†^ **U** = **I**.

A convenient approach is to employ the exponential parametrization of unitary matrices. Any unitary matrix can be expressed as the exponential of an anti-Hermitian matrix

14
$$U=e^{X}=\sum_{n=0}^{\infty }\frac{X^{n}}{n!}$$

where **X** ^†^ = – **X**.^11^ Therefore

15
$$U^{†}U={\left(e^{X}\right)}^{†}e^{X}=e^{X^{†}}e^{X}=e^{-X}e^{X}=I$$

For an anti-Hermitian matrix **X**, the spectral decomposition can be written as

16
$$X=V\Lambda V^{†}$$

where **V** is unitary and **Λ** is diagonal with purely imaginary eigenvalues

17
$$\Lambda =diag\left(i\lambda_{1},i\lambda_{2},...\right),,\lambda_{i}\in R$$

exponentiation then yields

18
$$U=Vdiag\left(e^{i\lambda_{1}},e^{i\lambda_{2}},...\right)V^{†}$$

which is manifestly unitary.

To generate random orbital rotations with a prescribed magnitude, we first construct a random anti-Hermitian matrix **X** and normalize it with its Frobenius norm given by

19
$$∥X{∥}_{F}=\sqrt{\sum_{i,j}|X_{ij}{|}^{2}}$$

This ensures $\sum_{k}{\mathrm{\lambda ̃}}_{k}^{2}=1$ , where $i{\mathrm{\lambda ̃}}_{k}$ are the eigenvalues of the normalized anti-Hermitian matrix **X̃**. The rotation amplitude is then controlled by introducing a scalar parameter *a*

20
$$U\left(a\right)=e^{a\mathrm{X̃}}=Vdiag\left(e^{ia{\mathrm{\lambda ̃}}_{1}},e^{ia{\mathrm{\lambda ̃}}_{2}},...\right)V^{†}$$

By tuning *a*, one can systematically adjust the extent of the orbital rotation while preserving unitarity.

The rotation of each space ​(core, active, virtual) and their mutual rotations can be controlled by partitioning the anti-Hermitian matrix **X** into blocks associated with the rotations between orbital subspaces given by

21
$$X=\left[\begin{array}{lll} X_{ii} & X_{ia} & X_{iv}\\ X_{ai} & X_{aa} & X_{av}\\ X_{vi} & X_{va} & X_{vv} \end{array}\right]$$

where the indices *i*, *a*, and *v* refer to core, active, and virtual orbitals, respectively. Diagonal blocks correspond to rotations within the same subspace, while off-diagonal blocks mix orbitals from different subspaces. The random orbital rotation calculations presented in this work ​(Section 4.1) only involve rotations within the active space. Thus, only the *X*_*aa*_ block has nonzero elements, while the other blocks are set to zero.

## Notes

The Supporting Information includes a PDF file containing: a figure of the CSF-ROHF orbitals for the N_2_ molecule that converged to a higher-energy local minimum, and a figure comparing the FCIQMC population dynamics of the SSG and full FCIQMC calculations for the P-cluster ​(PDF)
A ZIP archive is also provided, which contains representative input files and the molecular geometries of the systems presented in this paper. Details of the files can be found in readme.md in the archive ​(ZIP)
