# Supplementary material for: Spin-Adapted Restricted Open-Shell Hartree–Fock and Its Dynamic Correlation Extension
Source: J Chem Theory Comput. 2026 May 15;22(11):5523–38. doi: 10.1021/acs.jctc.6c00379 (PMC13255178; doi:10.1021/acs.jctc.6c00379)
Supplement: Supplementary file 1 [file ct6c00379_si_001.pdf]

Spin-Adapted Restricted Open-Shell Hartree–Fock and Its Dynamic Correlation Extension

Maru Song,*,† Luca Bonfirraro,† Ignacio Fdez. Galván,‡ Roland Lindh,‡ and Giovanni Li Manni*,†

†*Max Planck Institute for Solid State Research, 70569 Stuttgart, Germany*

‡*Department of Chemistry for Life Sciences, Uppsala University, P. O. Box 576, Uppsala 75123, Sweden*

E-mail: m.song@fkf.mpg.de; g.limanni@fkf.mpg.de

Supporting Information Available

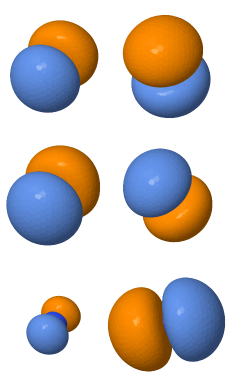

Figure S1: Six N_2 2p orbitals from a CSF-ROHF orbital optimization converged to a local minimum.

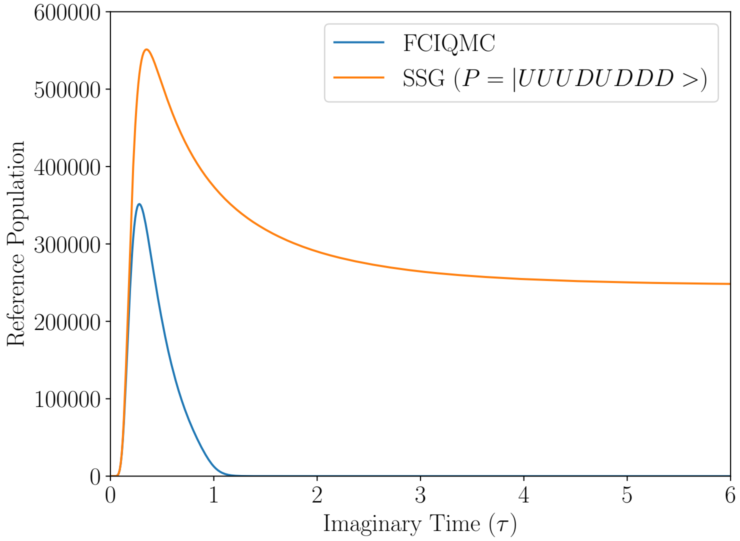

Figure S2: FCIQMC population dynamics for P-cluster CAS(48,40) calculations. Blue is the full FCIQMC run and orange is the single-CSF SSG run. Both runs used 1 million walkers and the initiator approximation was not used. The reference population curves clearly show that SSG can be stabilized with fewer walkers than full FCIQMC.
